# An epidemiological analysis of severe imported malaria infections in Sri Lanka, after malaria elimination

**DOI:** 10.1186/s12936-024-05014-w

**Published:** 2024-06-22

**Authors:** Shilanthi Seneviratne, Deepika Fernando, Rajitha Wickremasinghe, Sujai Senarathne, Pubudu Chulasiri, Nethmini Thenuwara, Champa Aluthweera, Iromi Mohotti, Shamila Jayakuru, Thilan Fernando, Anula Wijesundara, Rohini Fernandopulle, Kamini Mendis

**Affiliations:** 1 Anti Malaria Campaign, Ministry of Health, Colombo, Sri Lanka; 2 Department of Parasitology, Faculty of Medicine, University of Colombo, Colombo, Sri Lanka; 3 Faculty of Medicine, University of Kelaniya, Ragama, Sri Lanka; 4 Department of Parasitology, Faculty of Medical Sciences, University of Sri Jayewardenepura, Nugegoda, Sri Lanka; 5 Regional Malaria Office, Galle, Sri Lanka; 6 Colombo, Sri Lanka; 7 Faculty of Medicine, General Sir John Kotelawala Defense University, Ratmalana, Sri Lanka

**Keywords:** Severe malaria, Imported malaria, Sri Lanka, Prevention of re-establishment, Management, Delay in diagnosis, Travelers’ malaria

## Abstract

**Background:**

Imported malaria continues to be reported in Sri Lanka after it was eliminated in 2012, and a few progress to life-threatening severe malaria.

**Methods:**

Data on imported malaria cases reported in Sri Lanka from 2013 to 2023 were extracted from the national malaria database maintained by the Anti Malaria Campaign (AMC) of Sri Lanka. Case data of severe malaria as defined by the World Health Organization were analysed with regard to patients’ general characteristics and their health-seeking behaviour, and the latter compared with that of uncomplicated malaria patients. Details of the last three cases of severe malaria in 2023 are presented.

**Results:**

532 imported malaria cases were diagnosed over 11 years (2013–2023); 46 (8.6%) were severe malaria, of which 45 were *Plasmodium falciparum* and one *Plasmodium vivax*. Most severe malaria infections were acquired in Africa. All but one were males, and a majority (87%) were 26–60 years of age. They were mainly Sri Lankan nationals (82.6%). Just over half (56.5%) were treated at government hospitals. The average time between arrival of the person in Sri Lanka and onset of illness was 4 days. 29 cases of severe malaria were compared with 165 uncomplicated malaria cases reported from 2015 to 2023. On average both severe and uncomplicated malaria patients consulted a physician equally early (mean = 1 day) with 93.3% of severe malaria doing so within 3 days. However, the time from the point of consulting a physician to diagnosis of malaria was significantly longer (median 4 days) in severe malaria patients compared to uncomplicated patients (median 1 day) (p = 0.012) as was the time from onset of illness to diagnosis (p = 0.042). All severe patients recovered without sequelae except for one who died.

**Conclusions:**

The risk of severe malaria among imported cases increases significantly beyond 5 days from the onset of symptoms. Although patients consult a physician early, malaria diagnosis tends to be delayed by physicians because it is now a rare disease. Good access to expert clinical care has maintained case fatality rates of severe malaria at par with those reported elsewhere.

**Supplementary Information:**

The online version contains supplementary material available at 10.1186/s12936-024-05014-w.

## Background

Sri Lanka was endemic for malaria for centuries and has gone through devastating major outbreaks during the twentieth century [1–6]. Eliminating malaria and being certified by the World Health Organization (WHO) in 2016 was, therefore, considered a major public health achievement [5, 7–9]. At present, the country is actively engaged in preventing the re-establishment (PoR) of malaria amidst multiple challenges to sustain the malaria-free status [5, 10]. Travellers who acquire the disease overseas in malaria endemic countries, i.e. imported malaria, continue to present a constant risk of re-establishing malaria in the country in which malaria vectors are prevalent. Since the last reported indigenous case of malaria in 2012 until the end of 2023, Sri Lanka has reported 532 imported cases with, on average about 50 cases per year, one introduced case [11], and one transfusion induced case [12].

The key strategies implemented by the Anti Malaria Campaign (AMC) in the PoR phase include increasing awareness amongst physicians to suspect malaria in individuals with a travel history to a malaria endemic country so as to lead to early diagnosis and treatment, proactive case detection amongst high risk groups [13], providing chemoprophylaxis for travellers to malarious countries free of charge [14, 15] and vector surveillance and control [9]. These strategies implemented by the AMC have enabled Sri Lanka to sustain zero transmission of malaria and zero deaths due to malaria for a decade. Despite efforts one death due to imported malaria occurred in April of 2023 [16].

Clinical presentations of malaria are diverse, ranging from asymptomatic parasitaemia and uncomplicated illness to severe malaria with organ failure [17]. Nearly all deaths from severe malaria result from infections with *Plasmodium falciparum*, although *Plasmodium vivax, Plasmodium knowlesi, Plasmodium ovale* and *Plasmodium malariae* can also cause severe disease [18–22]. The WHO defines severe *P. falciparum* malaria as the occurrence of one or more of the following clinical features in the absence of an identified alternative cause and the presence of *P. falciparum* asexual parasitaemia, i.e. impaired consciousness, prostration, multiple convulsions, acidosis, hypoglycaemia, severe malarial anaemia, renal impairment, jaundice, pulmonary oedema, significant bleeding, shock, and hyperparasitaemia > 10% (> 400,000 parasites/μl) [23–25]. Hyperparasitaemia is associated with a high risk of fatality from falciparum malaria. In low transmission areas, mortality from falciparum malaria increases with parasite densities over 100,000 parasites/μl (i.e. approximately 2.5% parasitaemia), whereas in high transmission areas, higher parasite densities (> 800,000 parasites/μl; > 20% parasitaemia) are well tolerated [23]. Severe *P. vivax* and *P. knowlesi* malaria are defined as for falciparum malaria but with no parasite density threshold [24]. Progression to severe disease and death will depend on the infecting parasite species, the levels of innate and acquired immunity of the host, and the timing and efficacy of treatment [17].

In children severe malaria has a high mortality rate of 5%, compared to uncomplicated malaria, in which the mortality rate is 0.1% [26]. The risk of mortality due to malaria will increase as the total number of infecting organisms in the body increases. Thereby, in non-immune adults mortality increases steeply as peripheral blood parasite densities rise over 100,000/µL [25, 27]. A GeoSentinel analysis from 2003 to 2016 has reported that more than 30,000 malaria cases were reported annually among international travellers. Of the 5689 individuals included in the study, most (83%) were exposed in sub-Saharan Africa and 444 (8%) developed severe malaria, including 31 children. A case fatality rate of 0.2% (12 deaths) was recorded in the above study population [28].

This study aimed to give insight into severe malaria cases reported amongst imported malaria diagnosed and treated in Sri Lanka from 2013 to 2023. The last three cases of severe malaria reported in the country i.e. in 2023, are discussed in detail to illustrate the challenges encountered in diagnosing and managing severe imported malaria after the disease was eliminated.

## Methods

On notifying the AMC of a suspected case of malaria by a government or private hospital or a physician, confirmation of diagnosis is carried out by quality-assured microscopy at the central laboratory of the AMC or at the regional level by qualified Public Health Laboratory Technicians [29]. In every confirmed case of malaria a detailed history is obtained and recorded, including the history of the current illness, past history of malaria and travel history. From 2015 onwards details of how, when and where the patient sought health care for the current episode of malaria were recorded. Since 2013 information regarding each confirmed malaria patient has been entered into a National Malaria Register and the case database maintained at AMC Headquarters. Data with regard to imported malaria cases which included both severe and uncomplicated malaria reported in Sri Lanka from 2013 to 2023 were extracted from this database for further analysis.

A patient was classified as having severe malaria, if the individual had one or more features of clinical or laboratory evidence of vital organ dysfunction as defined by the WHO, in the absence of an identified alternative cause and the presence of a plasmodial asexual parasitaemia [23, 24].

The case data were analysed in relation to general characteristics of patients with severe malaria (i.e. age, sex, nationality, district of residence in Sri Lanka at the time of diagnosis, malaria species, country of origin of the infection), the type of health sector (private or public sector), the outcome of the disease and the patient’s health-seeking behaviour. The time intervals from arrival in Sri Lanka to onset of illness, from onset of illness to first contact with a physician, and from first contact with a physician to diagnosis of malaria were analysed in severe malaria patients and for comparison in all uncomplicated malaria patients reported during the same period of time. Analysis was performed using Microsoft Excel 365‐Microsoft Corporation Inc. Redmond, Washington USA. Version 2019 and the Statistical Package for the Social Sciences—SPSS Inc. IBM Corp. Armonk, New York, USA Version 21. Median test was used to compare the groups.

The recommended treatment regimen for severe malaria in Sri Lanka in accordance with the national malaria treatment guidelines is parenteral anti-malarial treatment with intravenous (IV) artesunate (2.4 mg/kg body weight) for a minimal duration of 24 h and until the patient reaches per os status, followed by a complete course of oral artemisinin-based combination therapy (ACT) using artemether-lumefantrine, as directly-observed treatment [13]. Severe malaria is considered a medical emergency and the patient is admitted to the intensive care unit (ICU) of a hospital and closely monitored with respect to vital signs, coma score (Glasgow coma score in adults or Blantyre coma score in children) and urine output. The attending Consultant Physician in collaboration with the Consultant Community Physicians of the AMC manages the patient. The AMC monitors the parasite density twice a day, i.e. in the morning and evening, until the parasitaemia reaches zero level. Following the required IV administration of artesunate, and completion of oral ACT, prior to discharge, the patients are given a single dose of 0.75 mg/kg of primaquine to eliminate gametocytes of *P. falciparum*. Severe *P. vivax* patients are treated in addition with primaquine for 7 days to prevent relapses. All confirmed malaria cases are followed up by repeated microscopic examination of blood on days 7, 14, 21, 28 and 42 for *P. falciparum* infections and day 7, 14, 21, 28, 42 and monthly for 1 year in *P. vivax* (considering the date of diagnosis as day 0) [13, 29].

Case investigation commences within 48 h of detection of a malaria patient and includes both parasitological and entomological surveillance. Travel cohorts of the malaria positive patient who lived in the same environment while overseas and returned to Sri Lanka are screened. Primary surveillance is carried out to screen persons living within a 1-km radius of the residence of the index case, and individuals the patient came in contact with if the patient stayed overnight elsewhere during the 2 weeks preceding the onset of symptoms to ensure that the index case did not acquire malaria from Sri Lanka. Primary surveillance is not recommended if the patient presents with signs and symptoms of malaria within 7 days of arrival in the country. Secondary surveillance is carried out in the above-mentioned cohort 2 weeks after diagnosis of malaria to ensure that the patient has not transmitted malaria in the community. Case based entomological surveillance, a reactive survey covering approximately 1-km radius from the location of the case is carried out within 48 h of diagnosing a malaria case. Further details regarding parasitological and entomological surveillance are described elsewhere [5, 9, 11, 12, 29].

## Results

A total of 532 imported malaria cases i.e. malaria infections which were acquired outside Sri Lanka, were diagnosed over the period 2013–2023. Of these, 256 individuals (48.1%) were *P. falciparum* infections (including two *P. falciparum* mixed infections), 215 (40.5%) were *P. vivax* and the rest were *P. ovale* (9.4%), *P. malariae* (1.9%) and *P. knowlesi* (0.19%). Approximately 90% (231/256) of *P. falciparum* cases including the two mixed infections were acquired in Africa, whereas 65% (140/215) of the imported *P. vivax* cases were acquired in India. Approximately 3/4ths i.e. 74% of all imported infections were diagnosed in Sri Lankans and of the imported *P. falciparum* infections, 84% were diagnosed in Sri Lankans, and the rest were in foreign nationals.

### Incidence of severe malaria and geographic origin

Of the imported malaria cases due to all species, 46 (8.6%) developed severe disease (Table 1). One case of severe malaria was due to *P. vivax*; the rest (n = 45) were due to *P. falciparum.* Forty five, i.e. 17.6% of *P. falciparum* infections developed severe disease. Except for two severe cases acquired in Guyana and India, respectively, the rest (n = 43) of the *P. falciparum* infections were acquired in African countries, as was the *P. vivax* case which was acquired in Madagascar. Of the severe *P. falciparum* cases acquired in Africa 46% were acquired in Eastern Africa, followed by 30% from Western Africa and 8% each in Central and North Africa.

**Table 1:** Incidence rate of imported severe Malaria in Sri Lanka 2013–2023

Year	Total number of imported malaria/*P.falciparum* cases	Number of severe malaria cases	Severe malaria cases as a proportion (%) of all malaria/of *P.falciparum* cases
2013	95/42	12	12.6/28.5
2014	49/20	4	8.1/20.0
2015	36/17	6	16.6/35.3
2016	41/18	9	21.9/50.0
2017	57/26	3	5.2/11.5
2018	47/15	4*	8.5/20.0
2019	53/24	0	0
2020	30/9	1	3.3/11.1
2021	25/12	1	4.0/8.3
2022	37/27	2	5.4/7.4
2023	62/46	4**	6.5/8.7
TOTAL	532/256	46	8.6/17.6

^*^Includes a case of *P.vivax* malaria acquired in Madagascar

^**^ Includes a fatal case of *P.falciparum* severe malaria acquired in Tanzania

### Profile of severe malaria patients

**Fig. 1:**
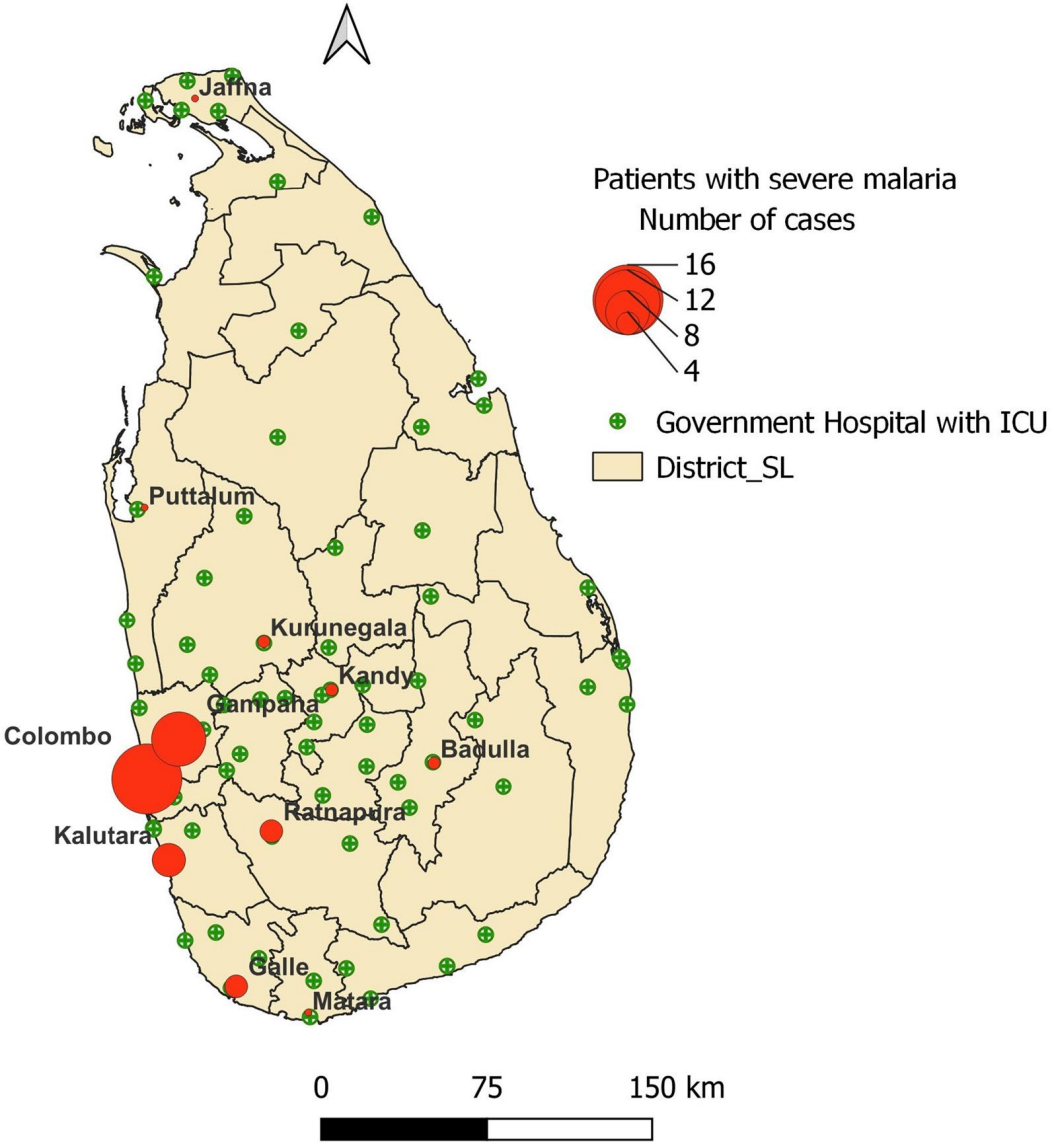
Map of Sri Lanka showing district boundaries, where severe malaria patients (2013–2023) were managed (red circles, diameter depicting number of patients) and the locations of government hospitals with ICU facilities

Age of the 46 severe malaria patients ranged from 17 to 67 years (mean: 41 years), 87% of them being between 26 and 60 years of age. Except for one female, all were males (Supplementary Table 1). A majority of individuals were Sri Lankan nationals (82.6%), with the remaining being foreign nationals from Ukraine, Australia, the UK, France, Indonesia, Lebanon, and Korea. All patients had sought healthcare from allopathic physicians, either Private General Practitioners (GPs) or physicians based in either government or private hospitals. Most (63%) of the patients with severe malaria were diagnosed and treated in the Western Province of the country (the highest number of patients from the Capital city of the country in the Colombo District, followed by Gampaha and Kalutara Districts) (Fig. 1 and Supplementary Table 1). Just over half the patients (n = 26/46, 56.5%) were treated at government-owned hospitals and the rest in private sector hospitals, with anti-malarial treatment commencing immediately after diagnosis. Apart from one severe malaria patient in whom the infection was fatal [16], all other patients made a complete recovery without sequelae.

### Time to diagnosis

**Fig. 2:**
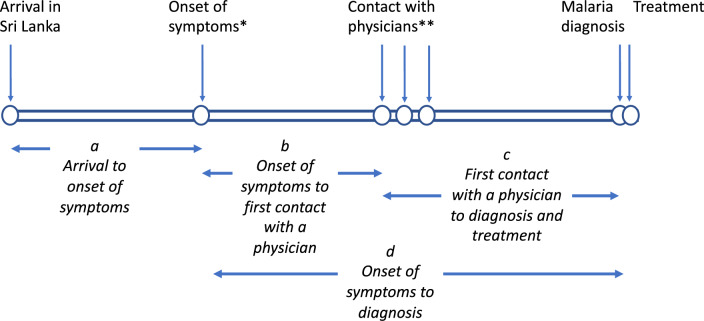
Schematic representation of events relating to imported malaria patients. * In a few symptoms would have arisen prior to arrival. ** Several physicians may have been consulted prior to a diagnosis of malaria being made

The time from arrival in the country to a diagnosis of malaria including the events between these was analysed in severe *P. falciparum* cases and for comparison in uncomplicated *P. falciparum* cases which were reported during the period of the study (Fig. 2).

#### Time from Arrival in Sri Lanka to onset of malaria illness

The average time taken from arrival to the onset of illness (Fig. 2a) in severe *P. falciparum* cases (n = 45) was 4 days (median: 4 days, IQR:1–7 days), and in uncomplicated falciparum malaria (n = 211) was 7 days (median:3 days, IQR:0–8 days) the difference not being statistically significant. In a few patients, symptoms began even before arrival in the country.

### Time from onset of illness to diagnosis of malaria

**Fig. 3:**
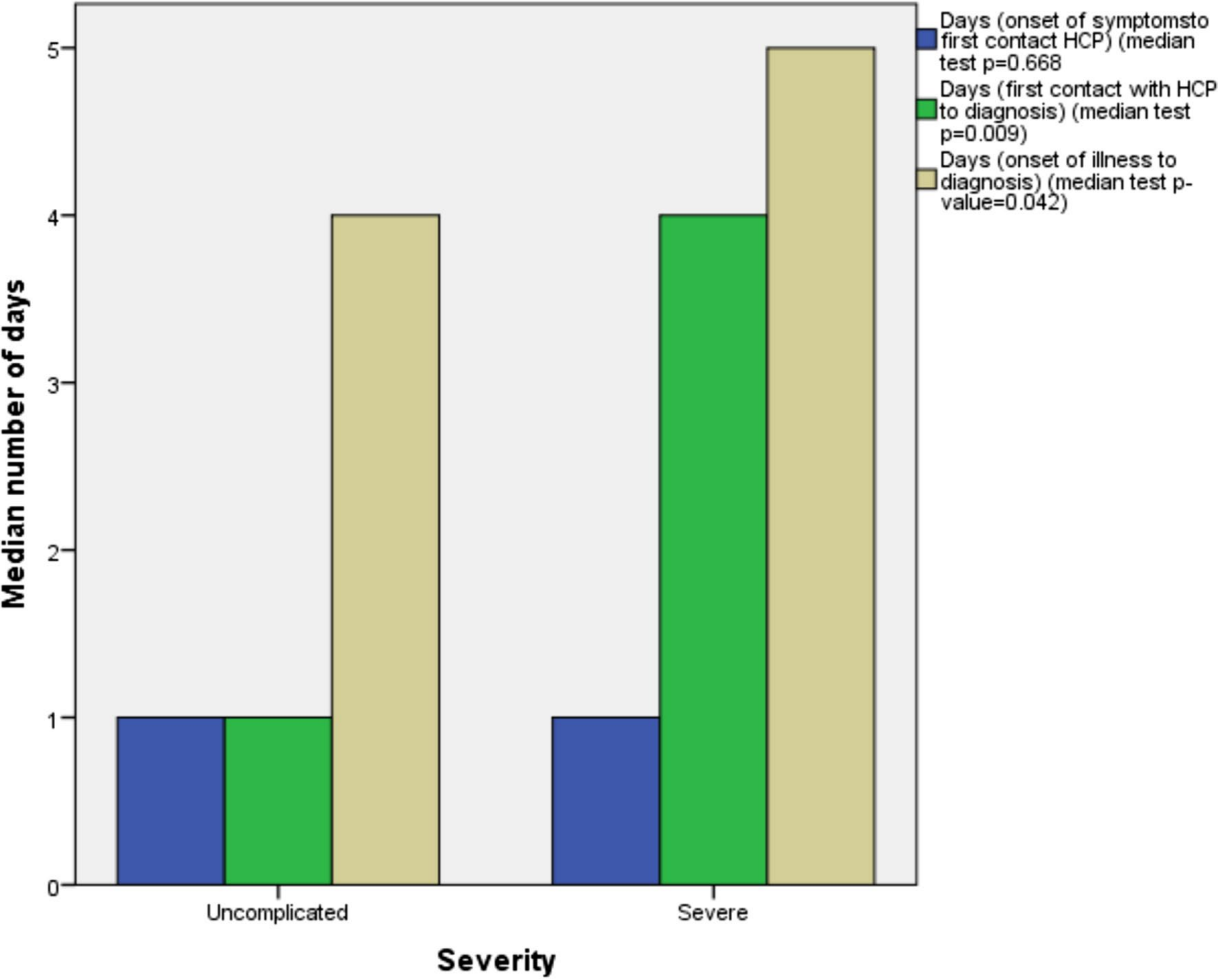
Time duration from onset of symptoms to first contact with a physician (blue), from first contact with a physician to diagnosis (green) and from onset of illness to diagnosis (brown) in uncomplicated falciparum (n = 165) and severe falciparum (n = 29) malaria cases diagnosed between 2015 and 2023

Data on these parameters (Fig. 3) were only available from 2015 onwards. There were 29 cases of severe malaria due to *P. falciparum* and 165 cases of uncomplicated falciparum malaria reported from 2015 to 2023. The average time taken from onset of illness to first contact with a physician (Fig. 2b) in patients who developed severe falciparum malaria was 1.7 days (median: 1 day, IQR:0–3 days) as compared to 2.7 days (median:1 day, IQR:0–3 days) in patients with uncomplicated falciparum malaria (median test; p = 0.668), the difference not being significant (Fig. 3). However, the average time taken from the first contact with a physician to diagnosis (Fig. 2c) in individuals who developed severe falciparum malaria was 4 days (median:4 days, IQR:1–6 days) as compared to 2.7 days (median:1 day, IQR:0–3 days) in those who developed uncomplicated falciparum malaria (Fig. 3), the difference being significant (median test; p = 0.009); the likelihood of being diagnosed as a severe case as compared to an uncomplicated case was 2.359 times higher after 4 days had lapsed between consulting a physician and diagnosis (95% CI of odds ratio: 1.036–5.373). The entire period from onset of illness to a malaria diagnosis (Fig. 2d) was also significantly longer in patients with severe malaria than in those with uncomplicated malaria (medians of 4 vs 5 days in uncomplicated and severe cases, respectively; p = 0.042) (Fig. 3).

Three cases of severe malaria diagnosed and treated in 2023 are described in detail below to illustrate some of the main challenges encountered in diagnosing malaria. All three patients were treated with the recommended anti-malarial drugs (as described in the methods section), and they made a complete recovery. These three cases were reported from previously non-endemic areas of Sri Lanka where the risk of onward transmission by *Anopheles culicifacies*, the primary vector of malaria in Sri Lanka was minimal.

### Case 1

A 37-year-old Sri Lankan male Seaman presented to the Emergency Treatment Unit (ETU) of a tertiary care Government hospital in the Southern Province of Sri Lanka with fever, chills and rigors, headache, body aches, and vomiting of one day’s duration on the 8th of August 2023. He had been travelling within the African Continent for 8 months (the last two ports of call being Cameroon and Gabon), and had returned to Sri Lanka 5 days prior to the onset of symptoms. Before admission, he had visited three General Practitioners (GP). The first two GPs had provided symptomatic treatment for a viral fever while four days after the onset of symptoms the 3rd GP requested a full blood count and advised admission suspecting dengue fever due to a low platelet count.

On admission to the ETU, the patient was conscious and rational. The Medical Officer of the ETU elicited the recent travel history to Africa and that he had not taken malaria chemoprophylaxis. On examination, the patient showed signs of respiratory distress with tachypnoea (20 breaths/minute) and was hypotensive (70/40 mmHg) with a high pulse rate (125 beats/minute). He had hepatosplenomegaly. Microscopy and a Rapid Diagnostic Test (RDT) for malaria were positive. The platelet count on admission was 6000/µL. Severe falciparum malaria was diagnosed based on the clinical presentation, rings and trophozoites of *P. falciparum* on microscopy with a parasite density 136,746/µL. The patient was transferred to the ICU and IV artesunate was administered beginning on the evening of admission (D0) along with a noradrenalin infusion at 0.2 µg/kg/min for hypotension. After three doses of IV artesunate 12 h apart the patient improved clinically and the parasite density declined. Oral ACT was started on D2 (third day after admission) and parasites cleared on D3. The patient was treated with 6 doses of oral artemisinin-based combination. During his 7 day stay in the ICU, vital signs were monitored and the patient was on a continuous oxygen supply. By D8 (nine days after admission), the patient was clinically stable and prior to discharge a single dose of primaquine was administered.

### Case 2

A 49-year-old Sri Lankan businessman who returned to Sri Lanka after a three month trip to Nigeria developed headache, body aches, and bloated abdomen 9 days after arrival. On the same day (i.e. on the 19th of August 2023), he visited a GP in his area of residence in the Southern Province, who prescribed treatment for gastritis. As the symptoms persisted, he visited the same GP five days later. During the second visit, the GP asked for a travel history to a malaria endemic country and requested a laboratory test for malaria. This patient had not been on malaria chemoprophylaxis. As the patient tested positive for *P. falciparum* by RDT and microscopy (ring stages with a parasite density of 80,400/µL) he was admitted to a District hospital in the Southern Province but as he was dyspnoeic with a respiratory rate of 44 breaths/min he was transferred immediately to a tertiary care hospital with ICU facilities in the same Province. On admission his platelet count was 30,000/ µL. On account of his respiratory distress in the presence of malaria parasites he was treated as severe malaria with IV artesunate for 24 h followed by 6 doses of oral ACT. At the end of the second day of admission (D1), the patient was clinically well, and the parasite count dropped to 96/µL. Parasites cleared by the third day of admission (D2). He was discharged from the hospital on D3 after administering a single dose of primaquine.

### Case 3

A 30-year-old Lebanese male employed in Nigeria as an Engineer for nearly three years, returned to Lebanon in August 2023. After a one month stay in Lebanon, he arrived in Sri Lanka for a vacation in September 2023. On the day following his arrival, he developed fever and headache and self-medicated to relieve symptoms. On the third day of illness (i.e. on the 23rd of September 2023), he sought treatment at a private hospital in the Uva Province of Sri Lanka. He requested that he be tested for malaria since he had experienced three episodes of malaria in the past and he had not been on malaria chemoprophylaxis while in Nigeria. He tested positive on RDT and blood smears were positive for ring/trophozoite stages *P. falciparum* with a parasite density of 518, 400/µL. On admission his platelet count was 42,000. The patient was clinically well, but due to the high parasite density he was admitted to the same hospital and managed as a case of severe falciparum malaria. With the first dose of IV artesunate the parasite count declined and reached zero on D3 (fourth day of admission). Following three doses of IV artesunate administered 12 hourly, he was treated with six doses of oral ACT.

Supplementary Table 2 provides the results of blood investigations of the three patients.

## Discussion

Imported malaria continues to be reported in Sri Lanka in travellers returning from malaria endemic countries. With an estimated 233 million cases of malaria being reported from the WHO African Region in 2022 [30], accounting for approximately 94% of the cases globally it is to be expected that most imported malaria cases diagnosed in non-endemic Western countries and countries which have eliminated malaria, such as Sri Lanka and Kyrgyztan, being acquired from Africa [31–37].

Over the eleven year period the study was carried out, 8.6% of all imported malaria patients and 17.6% of Imported *P. falciparum* cases in Sri Lanka progressed to severe disease, with the infection being fatal in one. Thus, case fatality of *P. falciparum* malaria being 0.39% (1/256) and of all imported malaria 0.18% (1/532). These figures are comparable to the proportion of severe malaria and deaths reported from other countries that have eliminated the disease. Trojánek et al*.*, in a 14 year retrospective single-centre descriptive study carried out between 2006 and 2019 in Prague, Czech, reported that 32 (15.8%) of the 203 individuals diagnosed with malaria presented with features of severe *P. falciparum* malaria (including one co-infection with *P. ovale*), with two deaths (case fatality rate of 1.0%) [31]. Spain reported 108 cases of imported malaria between April 2013 and April 2018 of which 13 (12%) developed severe malaria [32]. In the USA approximately 2000 persons were diagnosed with malaria in 2017 of which 14% developed severe disease with a case fatality rate of 0.3% [33]. In 2021, the UK reported 1,012 cases of imported malaria with 3 deaths (0.3% case fatality, all due to *P. falciparum* malaria) [34] and more recently, Giannone et al. [35] reported 8439 imported malaria cases in Switzerland over a thirty year period from 1990 to 2019 with 52 deaths, amounting to a case fatality rate of 0.62% [35].

The criteria for defining severe malaria have been regularly updated by the WHO since 1985 [25]. This was to ensure that a larger proportion of people with acute malaria will be managed appropriately with parenteral anti-malarials and supportive therapy to minimize sequelae and fatalities [17]. In this study patients were classified as severe based on the presence of any of the clinical features given in the current WHO definition of severe malaria with the presence of *P. falciparum* asexual parasitaemia [17, 23]. The *P. vivax* patient was classified as a severe malaria patient on the basis of respiratory distress and a blood pressure < 80 mmHg with evidence of impaired perfusion, two of the criteria of severe malaria as defined by the WHO. The two objectives identified in the National Strategic Plan of the Anti Malaria Campaign 2023–2027 are prevention of re-establishment of malaria and maintaining zero mortality due to malaria in Sri Lanka [38]. To achieve the latter, patients diagnosed with severe malaria are transferred to the closest hospital with an ICU and treated there. In remote hospitals where a Consultant Specialist Physician is available but no ICU facilities, IV artesunate is stocked in the ETU and the initial dose is given prior to transfer of the patient to a hospital with an ICU. In hospitals which have the services of a Specialist Consultant Physician and an ICU, IV artesunate is stored in a limited quantity of 3–4 vials to ensure that treatment is started immediately, until additional doses are mobilized from the Regional Malaria Office of that district or from another closest hospital. This minimizes drug wastage whilst also avoiding delays in treatment in a country where malaria infections are rare, and severe malaria cases are even more rarely encountered, and therefore expiry of drug stocks is a common occurrence. In this study, 43.5% of severe malaria patients were treated successfully in private sector hospitals, which are at par with the government sector hospitals in diagnosing and managing severe malaria. Given the location of Government hospitals (Fig. 1) and private sector hospitals (not shown) throughout the country access to Intensive care facilities in the public, or even the private sector is extremely good enabling patients to avail themselves of such services within a short span of time. Thus both government and private hospitals in Sri Lanka play a key role in managing patients with severe malaria and reducing sequelae and mortality. The staff of the AMC are closely engaged with the attending physician in patient management, providing data on parasite clearance, and guidance on anti-malarial treatment. In recent years, even the national malaria treatment guidelines published in Sri Lanka were updated for the treatment of patients with a parasite density between 100,000 and 200,000 (2.5–5.0%). Even though such individuals do not fall within the category of severe malaria, they are to be treated with a single dose of IV artesunate followed by oral ACT to rapidly reduce the parasite load. This was to reduce the risk of them progressing to severe malaria if parasite clearance is delayed [13]. Overall, it appears that good access to expert clinical care has allowed case fatality rates of severe malaria to be maintained at par with those reported from other malaria non-endemic countries.

To investigate if delays in diagnosis contributed to the severity of malaria infections the time durations between the onset of illness to the point of consulting a physician for the first time and ultimately to a diagnosis and treatment of malaria were analysed, the latter being started immediately after diagnosis in all patients. These time periods were compared between *P. falciparum* patients with severe malaria and those with uncomplicated malaria reported over the same period of time. All malaria patients, both severe and uncomplicated had consulted a physician within a few days of the onset of symptoms (average of 1.7 and 2.7 days respectively) there being no significant difference between the two categories. However, from the point of consulting a physician onwards it has taken significantly longer for a malaria diagnosis to be made in patients who eventually developed severe malaria. Overall, severe malaria patients also took significantly longer from the onset of symptoms to being diagnosed with malaria than uncomplicated patients. The data suggests that up to about 5 days from the onset of malaria symptoms, malaria infections in travellers are at a low risk of progressing to severe malaria. Furthermore, the results show that patients themselves seek healthcare early enough to be within this safe period. However, thereafter, the risk increases considerably with delays in diagnosis on the part of the physicians being consulted. This has important implications for the country to achieve its aim of maintaining zero malaria deaths.

The only death of an imported severe malaria patient since malaria was eliminated occurred in April of 2023 and associated with a delay in diagnosis and may have been avoidable [16]. The patient had sought medical attention immediately upon arrival in Sri Lanka with symptoms of acute but uncomplicated malaria, but the disease was not suspected by the first nor two other GPs whom he consulted subsequently in the following 4 days. Five days later, he was admitted in a critical condition to a major private hospital where, though a malaria microscopy report was made available following admission, the patient died soon after before any medication could be administered [16]. Delay in diagnosis of malaria in patients continues to remain a major challenge in Sri Lanka, where the disease is rare. Even in the current study, more than 90% of the patients who progressed to severe malaria contacted a physician within 3 days of onset of symptoms but in more than half of the patients a diagnosis of malaria was made later than 3 days after the first consultation.

Post-malaria elimination, the failure of physicians to suspect and test for malaria even in patients with typical febrile symptoms of malaria is being encountered repeatedly in Sri Lanka, the disease being infrequent [39–41]. Other more prevalent febrile illnesses, such as dengue, take precedence in the differential diagnosis of patients presenting with fever, and the rapid increase in the incidence of dengue cases over the past few years has contributed to it. In 2023 alone 89,799 dengue cases and 62 deaths were reported in the country (Personal communication, Dr. S. Samaraweera, Director, National Dengue Control Unit, Sri Lanka). For example, Case 1 presented with typical signs and symptoms of malaria but was admitted to hospital by the GP on suspicion of dengue due to a low platelet count. It has been previously reported on how low platelet counts in malaria patients constitute a red-herring, creating a false trail to a dengue diagnosis, it being a leading cause of delays in malaria diagnosis [42]. Case 2 who presented with more atypical clinical features of headache, body aches, and a bloated abdomen and no fever, was treated for gastritis at the initial visit to a GP.

Passive case detection still remains the main strategy for malaria diagnosis in Sri Lanka, accounting for over 90% of cases diagnosed [40, 41, 43]. It is supplemented by Proactive Case Detection and Reactive Case Detection in particular situations [5]. To reduce the time to a malaria diagnosis, awareness programmes on malaria are being carried out for physicians by the AMC through continued collaboration with professional medical associations and Colleges, the key slogan being “Suspect malaria if an individual presents with fever and travel history to a malaria endemic country”. The AMC also carries out an active social media campaign targeting both travellers and healthcare providers to enhance awareness of imported malaria and promote early diagnosis. As the national case management guidelines may not reach all GPs who are solely engaged in private practice, the AMC, through the College of General Practitioners and the Independent Medical Practitioners Association organizes clinical lectures in the districts. National malaria treatment guidelines providing criteria to suspect malaria, have been sent by the Director General of Health Services to every government and private hospital [13].

Prevention of malaria in Sri Lankan travellers is yet another strategy being implemented by the AMC to reduce incidence, morbidity and mortality due to imported malaria. Studies have shown that malaria chemoprophylaxis is well tolerated by travellers with a protective efficacy of 100% amongst those who complied [14]. The AMC provides malaria chemoprophylaxis free-of-charge to any traveller destined for a malaria endemic country. Yet, despite the wide availability of anti-malarial chemoprophylaxis at the AMC Headquarters and Offices of the Regional Malaria Officer travellers to malaria endemic countries rarely obtain these medicines, and even when they do, adherence to the prophylaxis regime has been poor [14, 44]. Between 2015 and 2023, of the 30 patients who developed severe malaria, only five had obtained prophylaxis and they too had not taken it as advised (Supplementary Table 1). In addition, long-lasting impregnated nets are provided to security forces personnel travelling to malaria endemic countries free of charge to be taken with them on their journey.

Many Sri Lankans who acquire imported malaria had travelled to Africa for occupational purposes, key identified groups being security forces personnel proceeding to South Sudan and Central African Republic on United Nations peacekeeping missions and gem traders who travel to Tanzania and Madagascar [5, 41]. These individuals are made aware of the risk of malaria, the preventive measures to be taken and the need to get tested for malaria once they return to the country, by programmes organized by the AMC.

This study on severe imported malaria in Sri Lanka provides evidence that delayed diagnosis of imported malaria beyond the fifth day since the onset of symptoms, and a delay in diagnosis of more than 4 days from the first contact with a healthcare provider significantly increases the risk for developing severe malaria in those who are prone to develop severe malaria. The study also shows that a vast majority of malaria patients seek treatment within this safe period, and that it is delays on the part of physicians to diagnose malaria thereafter that increases the risk of severe malaria. These findings suggests that in order to maintain zero mortality due to imported malaria, the primary focus of advocacy for early diagnosis of malaria should be physicians, who, due to the rarity of malaria infections in the country fail to suspect and test for the disease. We have previously reported that in 9% of malaria infections in Sri Lanka a diagnosis was made because of a self-request by the patient for a malaria diagnostic test [41], as was the case with patient 3 above. The message to potential travellers to and from malaria endemic countries should be to request a malaria diagnosis when they consult a doctor, rather than merely to seek treatment early, because, as this study shows, even those who developed severe malaria did seek treatment early enough to have prevented the progression to severe malaria.

## Conclusions

Severe imported malaria, due mainly to *P. falciparum* infections continues to be reported from Sri Lanka among travellers after the disease was eliminated. Data presented here point strongly to delayed diagnosis of malaria beyond day 5 of the onset of symptoms, and 4 days after contact with a healthcare provider as increasing the risk for severe malaria in travellers by more than twofold. Most patients with imported malaria seek healthcare for malaria infections early enough to prevent severe disease. However, it is the failure to suspect and therefore diagnose malaria by healthcare providers that delays treatment. This happens against the backdrop of an extremely low malaria disease burden, and a high incidence of other febrile diseases, such as dengue, which leads to malaria being overlooked in the differential diagnosis of febrile illnesses. Efforts to reduce such delays in malaria diagnosis will have impact on reducing severe malaria and death in travellers.

## Recommendations

Recommendations which accrue from this study to prevent severe malaria in countries which have eliminated malaria are:

1. Physicians to be regularly alerted to the need to seek a travel history to malaria endemic countries in febrile patients, and test for malaria accordingly, to ensure early diagnosis and treatment of malaria.
2. Malaria rapid diagnostic tests and IV artesunate to be made available in all intensive care units of tertiary hospitals in areas which are at risk of importation.
3. In all patients with impaired consciousness and a travel history to endemic countries testing for malaria is performed as a routine practice.
4. Staff of intensive care units to be updated on these guidelines on a regular basis.

### Supplementary Information

Additional file 1.

Additional file 2.

## Data Availability

The datasets generated and/or analysed in this publication are not publicly available due to the fact that they belong to the Ministry of Health, Sri Lanka. Clarifications regarding data can be made through Dr. Champa Aluthweera, Director of the Anti Malaria Campaign, Sri Lanka who is an author of this publication.

## Abbreviations

ACT: Artemisinin-based combination therapy
AMC: Anti malaria campaign
ETU: Emergency treatment unit
GP: General practitioner
ICU: Intensive care unit
IV: Intravenous
PoR: Prevention of re-establishment
RDT: Rapid diagnostic tests
WHO: World Health Organization
