# Supplementary material for: An epidemiological analysis of severe imported malaria infections in Sri Lanka, after malaria elimination
Source: Malar J. 2024 Jun 22;23:195. doi: 10.1186/s12936-024-05014-w (PMC11193279; doi:10.1186/s12936-024-05014-w)
Supplement: Supplementary file 1 — Additional file 1. [file 12936_2024_5014_MOESM1_ESM.docx]

**Supplementary Table 1: General characteristics of patients with severe malaria from 2013-2023**

| **Characteristic** | **Number** | **Percentage (%)** |
| --- | --- | --- |
|  |  |  |
| **Age category (years)** | | |
| 15-25 | 5 | 10.9 |
| 26-60 | 40 | 86.9 |
| >60 | 1 | 2.2 |
| **Sex** | | |
| Male | 45 | 97.8 |
| Female | 1 | 2.2 |
| **Nationality** | | |
| Sri Lankan | 38 | 82.6 |
| Foreigner | 8 | 17.4 |
| **Purpose of travel** | | |
| Migrant workers (Sri Lankan) | 11 | 23.9 |
| Security forces personnel on UN Missions or training | 10 | 21.8 |
| Gem trade (craft workers) | 10 | 21.8 |
| Fisheries industry | 5 | 10.9 |
| Seaman | 3 | 6.5 |
| Arrived in Sri Lanka as a tourist | 3 | 6.5 |
| Student | 2 | 4.3 |
| Migrant workers (foreign) | 2 | 4.3 |
| **District of diagnosis** | | |
| Colombo | 14 | 30.4 |
| Gampaha | 10 | 21.8 |
| Kalutara | 5 | 10.8 |
| Others | 17 | 37.0 |
| **Type of hospital treated at** | | |
| Government hospital | 26 | 56.5 |
| Private hospital | 20 | 43.5 |
| **Chemoprophylaxis (n=30)** | | |
| Yes, regular | 0 |  |
| Yes. Irregular | 5 | 16.7 |
| No | 25 | 83.3 |
