# Supplementary material for: An epidemiological analysis of severe imported malaria infections in Sri Lanka, after malaria elimination
Source: Malar J. 2024 Jun 22;23:195. doi: 10.1186/s12936-024-05014-w (PMC11193279; doi:10.1186/s12936-024-05014-w)
Supplement: Supplementary file 2 — Additional file 2. [file 12936_2024_5014_MOESM2_ESM.docx]

**Supplementary Table 2: Blood investigations of the three case studies on admission (D0)**

| **Parameter** | **Case 1** | **Case 2** | **Case 3** |
| --- | --- | --- | --- |
| Parasitaemia | 136,746/µL | 80,400/µL | 518, 400/µL |
| Leukocytes | 2510/µL | 9320/µL | 4540/µL |
| Neutrophils | 84% | 81.9% | 62.2% |
| Lymphocytes | 12% | 10.2% | 19. 9% |
| Eosinophils | 3% | 0.2% | 0.6% |
| Platelets | 6000/µL (increased to 66,000/µL by day 5) | 30,000/µL | 42,000/µL |
| Packed Cell Volume | 46.5% | 37.9% | 32.3% |
| Haemoglobin | 15.9 g/dL | 13.4 g/dL | 11.5 g/dL |
| Alanine Transaminase | 124 IU/L | 38 IU/L | 208 IU/L |
| Aspartate transaminase | 79 IU/L | 34 IU/L | 293 IU/L |
| Serum creatinine | 170 µmol/L | 81 µmol/L | 194.7 µmol/L |
| c-reactive protein | 21.8 mg/dL | 196 mg/dL | 194.7 mg/dL |
| Blood urea | 39 mg/dL | - | - |
| Serum bilirubin | 44 µmol/L | 64.1 μmol/L | 17.1 μmol/L |
| Urine Full Report | Normal | Normal | Normal |
